# Changes in serotype prevalence of *Streptococcus pneumoniae* in Southampton, UK between 2006 and 2018

**DOI:** 10.1038/s41598-022-17600-6

**Published:** 2022-08-03

**Authors:** David W. Cleary, Jessica Jones, Rebecca A. Gladstone, Karen L. Osman, Vanessa T. Devine, Johanna M. Jefferies, Stephen D. Bentley, Saul N. Faust, Stuart C. Clarke

**Affiliations:** 1 Faculty of Medicine & Institute for Life Sciences, University of Southampton, University Hospital Southampton Foundation NHS Trust, Southampton, UK; 2 NIHR Southampton Biomedical Research Centre, University Hospital Southampton Foundation NHS Trust, Southampton, UK; 3 Parasites and Microbes, Wellcome Sanger Institute, Wellcome Genome Campus, Hinxton, UK; 4 Department of Biostatistics, Institute of Basic Medical Sciences, University of Oslo, Oslo, Norway; 5 NIHR Southampton Clinical Research Facility, University Hospital Southampton Foundation NHS Trust, Southampton, UK; 6 Global Health Research Institute, University of Southampton, Southampton, UK

**Keywords:** Conjugate vaccines, Infectious-disease epidemiology

## Abstract

*Streptococcus pneumoniae* continues to cause significant disease burden. Whilst pneumococcal conjugate vaccines (PCV) have substantially reduced this burden, serotype replacement partially negates this success due to increased disease associated with non-vaccine serotypes (NVTs). Continued surveillance is therefore essential to provide crucial epidemiological data. Annual cross-sectional surveillance of paediatric pneumococcal carriage was started in Southampton, UK following PCV7 roll-out in 2006. Nasopharyngeal swabs were collected from children < 5 years old each winter (October to March) from 2006/07 and for each consecutive year until 2017/18. Pneumococcal serotype was inferred from whole genome sequencing data. A total of 1429 (32.5%) pneumococci were isolated from 4093 children. Carriage ranged from 27.8% (95%CI 23.7–32.7) in 2008/09 to 37.9% (95%CI 32.8–43.2) in 2014/15. Analyses showed that carriage increased in children aged 24–35 months (*p* < 0.001) and 47–60 months (*p* < 0.05). Carriage of PCV serotypes decreased markedly following PCV7 and/or PCV13 introduction, apart from serotype 3 where the relative frequency was slightly lower post-PCV13 (pre-PCV13 n = 7, 1.67%; post-PCV13 n = 13, 1.27%). Prevalence of NVTs implicated in increased disease was low with 24F (n = 19, 1.4%) being the most common followed by 9N (n = 11, 0.8%), 8 (n = 7, 0.5%) and 12F (n = 3, 0.2%).

## Introduction

As a frequent coloniser of the upper respiratory tract, *Streptococcus pneumoniae*, the pneumococcus, continues to cause significant global burden of both invasive and non-invasive diseases. These include diseases such as meningitis, sepsis, pneumonia and otitis media (OM)^1^. The most recent estimates suggest that ~ 300,000 deaths of children under the age of five occur each year globally, a mortality rate of 45/100,000, with the majority a consequence of pneumonia^1^.

The introduction of pneumococcal conjugate vaccines (PCVs) into national immunisation programmes in the early 2000s substantially reduced both mortality and morbidity from this bacterium. PCVs use one of the primary virulence determinants of the pneumococcus, its polysaccharide capsule, of which there are now 100 defined types^2^. Initial PCV formulation targeted those serotypes causing the greatest disease burden. Continued surveillance has since seen the formulation of PCVs expand to include serotypes which subsequently increased in disease prevalence. In the USA, modelling showed that over 282,000 invasive pneumococcal disease (IPD) cases and ~ 3000 deaths have been averted, with 97 million visits to health-care providers for OM also avoided^3^. A similar benefit was also observed in England and Wales following the introduction of the seven-valent PCV (PCV7) in 2006. PCV7 targeted serotypes 4, 14, 18C, 19F, 23F, 6B and 9V, and was replaced in 2010 by a thirteen–valent PCV (PCV13) containing an additional six serotypes: 1, 3, 5, 19A, 6A and 7F. Accounting for both direct protection and herd immunity in non-vaccine recipients of all age groups^4, 5^ almost 40,000 cases of IPD have been prevented in England and Wales in the eleven years after PCV7 introduction^6^.

Overall carriage prevalence of pneumococci has remained largely unaffected despite PCV introductions and instead the phenomenon of serotype replacement has occurred^7, 8^. Here, non-vaccine serotypes (NVTs) inhabit the vacant ecological niche that remains following the removal of circulating VT pneumococci. Although NVTs typically have less invasive potential and overall IPD incidence is lower post-PCV, serotype replacement is nevertheless problematic as it partially negates the success of PCVs due to increases in disease associated with NVTs. For example, in England and Wales worrying increases in Serotypes 8, 12F and 9N have been observed in IPD surveillance data^6^. Globally 15A and 23B have been also been flagged as serotypes of concern^9^. Contrasts such as this highlight observed differences in serotype replacement when looking at data from individual countries. The possible reasons for this have been expertly reviewed recently^10^. Leaving aside study design and the way in which disease is reported, likely drivers for these differences include the pneumococcal population ecology pre-vaccination and the prospect that cross-sectional carriage studies are unable to accurately identify rarer NVT serotypes that go on to be a significant proportion of IPD. In this situation those same serotypes are hypothesised to have a high case to carrier ratio. Regardless of these issues, continued surveillance is therefore essential to provide crucial epidemiological data relating to the ever-evolving landscape of circulating pneumococci.

During the introduction of PCV7 in the UK, we started an annual surveillance study of carriage in children aged < 5 years in the Southampton area. This unique study has since yielded important data regarding asymptomatic carriage of this significant pathogen in children < 5 years of age. This has aided our understanding of both vaccine efficacy for current PCVs whilst also improving our understanding of the relationship between serotype replacement in the broader national context of invasive disease^7, 8, 11^. Here we report the carriage epidemiology of pneumococcal serotypes over a period of twelve winter seasons, from 2006/07 to 2017/18. We found potentially important increases in carriage prevalence in older children particularly. Additionally, whilst carriage of VTs was substantially diminished, there was a continued low-level circulation of serotypes 3 and 19A.

## Methods

### Ethical approval

The study was approved by the UK National Health Service (NHS) Research Ethics Service (06/Q1704/105 and 14/NS/1064). All methods and research practises outlined below were performed in accordance with relevant regulations which included the taking of informed consent from the legal guardians of all participants.

### Paediatric population

The study site, Southampton General Hospital, is administered by University Hospital Southampton (UHS) NHS Foundation Trust, which serves a population of approximately 1.9 million in Southampton and South Hampshire. The resident population of Southampton is ~ 250,000 with ~ 16,000 being children aged < 5 years. At 6% this is in keeping with the national average^12^ although we note that children would inevitably have also been recruited from outside the City of Southampton. Southampton is an ethnically diverse city with 78% of residents being White British or Irish in the 2011 census; a proportion that has likely decreased given that in 2017/18 nearly 40% of live births were of non-White British or Irish ethnicity^12^.

### Nasopharyngeal swab samples and laboratory processing

Nasopharyngeal swabs were collected from children aged < 5 years each year commencing in the winter (October to March) of 2006/07 and for each consecutive year until 2017/18. Parents/guardians were approached for informed consent either prior to or following their child’s appointment in an outpatient department of Southampton General Hospital. Aside from age, the only other exclusion criterium was that only one child per family was swabbed and that child was swabbed only once. Each year the target for isolation was n = 100 pneumococci. Assuming a low carriage prevalence of 10%, this would allow the detection ~ 50% relative reduction with 80% power at a 5% significance level. Nasopharyngeal Rayon tipped Transwabs (Medical Wire, Corsham, UK) in charcoal Amies media were used for swabbing and then plated onto Columbia Colistin Naladixic Acid agar (CNA; Oxoid, Basingstoke, UK) within 9 h of swabbing. Confirmation of presumptive *S. pneumoniae* was done on 5% blood Columbia Blood Agar (CBA; Oxoid, Basingstoke, UK) using optochin sensitivity indicated by a ⩾14 mm diameter inhibition zone around the disc (Thermo Scientific™, Loughborough, UK). Only one colony of *S. pneumoniae* per participant swab was selected for further analysis. Between 2006/07 and 2011/12 this was done in the Health Protection Agency Southampton Laboratory (now part of Public Health England) and from 2012/13 by technical staff in our research group.

### Questionnaire

In the winter of 2010/11, a questionnaire was introduced for the parent/guardian of each participant to complete. This questionnaire captured such information as vaccine status (Prevenar, Bexsero, Fluenz and whether the child was on schedule for routine paediatric vaccinations), recent respiratory illness (cold, ear infection, flu-like illness, sore throat or chest infection within the preceding 30 days), and antibiotic use within the preceding 30 days, with type if known.

### Serotyping

Isolates from skim milk, tryptone, glucose, and glycerin (STGG) stocks were cultured on CNA plates and incubated overnight at 37 °C in 5% CO_2_ prior to DNA extraction. Extraction was carried out using QIAamp® DNA mini kit (Qiagen, Hilden, Germany) according to the manufacturer's instructions. The DNA extracts were sent to the Wellcome Sanger Institute (WSI) for whole genome sequencing (WGS) using Illumina HiSeq or 10X platforms generating initially 2 × 75 bp and later 2 × 100 bp paired-end reads from libraries prepared using TruSeq chemistry. Pneumococcal serotype was inferred using PneumoCaT version 1.0^13^.

### Statistical analysis

All statistical analysis was done in R version 3.6.0 (2019-04-26) using RStudio version 1.2.1335^14, 15^ with graphics built using the grammar of graphics package, ggplot^15^. Participants characterised by recruitment in the PCV7 or PCV13 era were defined as having swabbing dates of 01/01/2006–30/06/2010 inclusive and 01/07/2010 to the end of the study period respectively. To evaluate the impact of PCV dose on pneumococcal carriage a likely dose number was assigned using the child’s age i.e., those > 1.0 month and ≤ 3.9 months were labelled as having one dose, those ≥ 4.0 months and ≤ 11.9 months were labelled as two doses, and those aged ≥ 12.0 months but ≤ 24 months were assumed to have received three doses. An upper age limit was used to minimise confounding effects of increasing pneumococcal carriage with age. When evaluating doses, particularly for comparisons of PCV7 versus PCV13, we excluded year one (2006/07) and year five (2010/11) to avoid confounding effects of catch-up and exclude those whose vaccinations might have spanned the period of PCV13 roll-out. Chi-squared test for trend in proportions was done using the prop_trend_test() in the R package rstatix. Simpsons index of diversity was computed using the diversity() function from the R package vegan^16^. Odds ratios based on multivariable logistic regression analysis and forest plots were generated using the R package finalfit() and glmulti() where the dependent variable was carriage of *S. pneumoniae*, the explanatory variables a character list derived from questionnaire data, and the random effect the year of the study.

## Results

**Figure 1:**
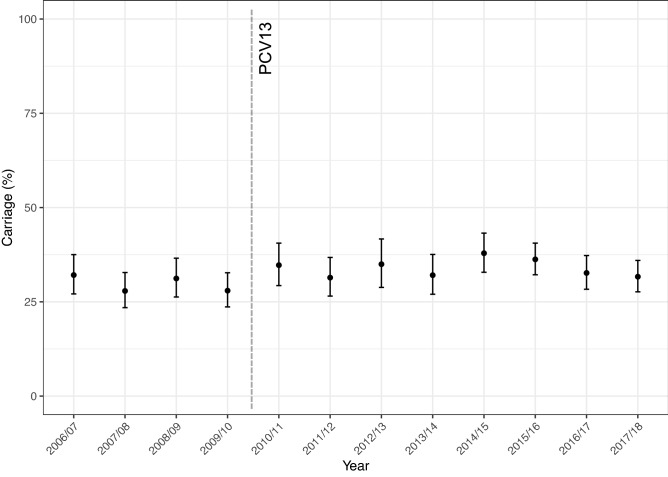
Carriage prevalence (%) of *S. pneumoniae* (all serotypes) in each year of the study. Overall carriage has remained consistent at an average of 32.5%, ranging from 27.8% in 2008/09 to 37.9% in 2014/15. Introduction of PCV13 is shown by the vertical line. Error bars represent 95% CI.

Between the winters of 2006/07 and 2017/18, from a total of 4409 study participants, 4393 children for whom age was accurately recorded as < 5 years provided NP swabs (n = 4393) for microbiological testing. Demographics of the study population are given in Supplementary Table 1. A breakdown of colonisation prevalence by age group per year with colonisation status is shown in Supplementary Fig. 1. A total of 1429 (32.5%) swabs yielded pneumococci. Pneumococcal carriage for each study year is shown in Fig. 1. Carriage ranged from a low of 27.8% (95%CI 23.7–32.7) in Year 3 (2008/09) to a high in Year 9 (2014/15) of 37.9% (95%CI 32.8–43.2).

**Figure 2:**
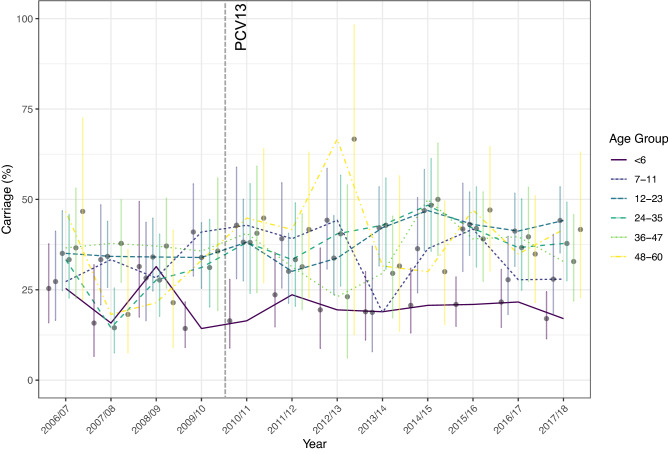
Carriage prevalence (%) of *S. pneumoniae* (all serotypes) by age group and year. Introduction of PCV13 is shown by the vertical line. Error bars represent 95% CI. Statistically significant increases in carriage in those aged 24–35 (*p* < 0.001) and 48–60 (*p* < 0.05) months old were shown by Chi-squared test for changes in trends of proportion.

**Figure 3:**
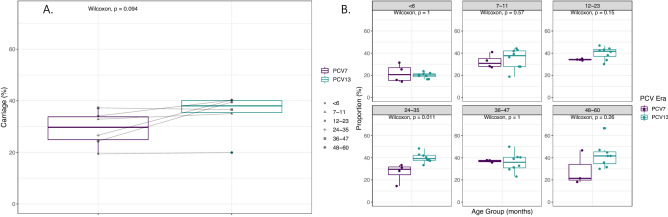
Comparison of overall pneumococcal carriage prevalence (%) by PCV era by all ages (**A**). and within age groups (**B**). Overall, no statistically significant difference was found between the PCV7 and PCV13 era, apart from in those aged 24–35 months, in whom a significant increase in carriage in the PCV13 era was observed (*p* < 0.05).

Pneumococcal carriage in age groups is shown in Fig. 2. Participants were divided into age strata as follows: < 6 months (n = 1009, 23.0%), 7–11 months (n = 644, 14.6%), 12–23 months (n = 1148, 26.1%), 24–35 months (n = 741, 16.9%), 36–47 months (n = 567, 12.9%), and 48–60 months (n = 284, 6.5%). Trend analyses showed that carriage increased over time in children aged 24–35 months (*p* < 0.001) and 47–60 months (*p* < 0.05). We next questioned whether these changes in carriage were attributable to the replacement of PCV7 with PCV13 between years four and five (2009/10 and 2010/11) (Fig. 3). As shown in Fig. 3A, there was no significant increase in carriage when all participants were grouped into either a PCV7 or PCV13 era (*p* = 0.094). However, when examining the age groups individually (Fig. 3B) here again a significant increase in carriage in those aged 24–35 months old in the PCV13 compared to PCV7 era was noted (*p* = 0.004).

**Figure 4:**
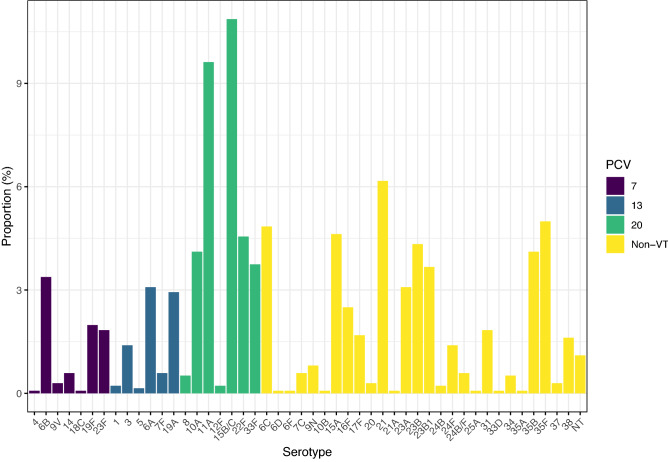
Serotype proportion for all *S. pneumoniae* isolated between 2006/07 and 2017/18. Serotypes are colored according to the PCV in which they are currently included (PCV13), previously included (PCV7) or included in newly licensed formulations (PCV20). Serotypes not in a PCV are shown in yellow.

The distribution of serotypes carried, according to their inclusion in PCV7 or PCV13, is shown in Fig. 4.

Those serotypes that will be targeted as part of PCV20, which is anticipated to be part of a childhood schedule in the future is therefore also shown. The most prevalent PCV7 serotypes were 6B (n = 46, 3.4%), 19F (n = 27, 2.0%) and 23F (n = 25, 1.8%). From PCV13, 6A (n = 42, 3.1%) and 19A (n = 40, 2.9%) were the most isolated. The NVT serotypes 15B/C and 11A were the most frequently isolated overall at 10.9% (n = 148) and 9.6% (n = 131) respectively.

**Figure 5:**
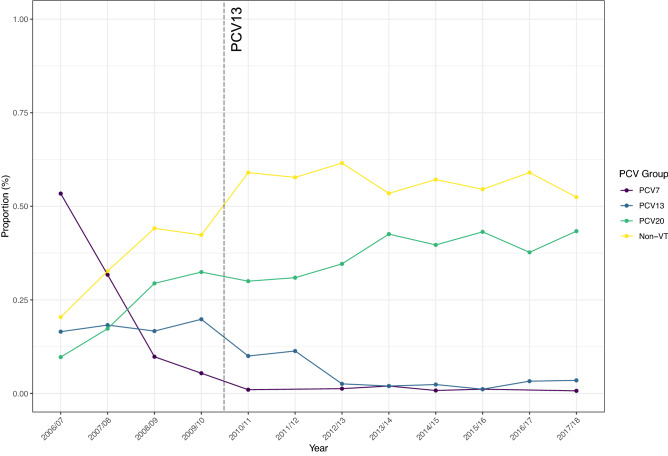
Prevalence of serotypes grouped by inclusion in PCVs, noting the reduction in PCV7 serotypes post-PCV7 from > 50% (purple) and of PCV13 serotypes (blue) from a high of ~ 20% post-PCV13. Serotype replacement with NVTs, here split to illustrate those that will be targeted by PCV20, equates to 95% of carriage in 2017/18.

No significant change in serotype diversity, as measured using Simpsons 1-D, was observed. Values ranged from 0.90 in 2006/07 to 0.94 in 2008/09 and 2009/10, with a mean of 0.93. When grouped in relation to PCV (Fig. 5), the reduction in PCV7 and PCV13 serotypes following PCV7 and PCV13 introduction is clear, with the concomitant increase in NVT serotypes, which in 2017/18 equated to > 95% of carriage.

**Figure 6:**
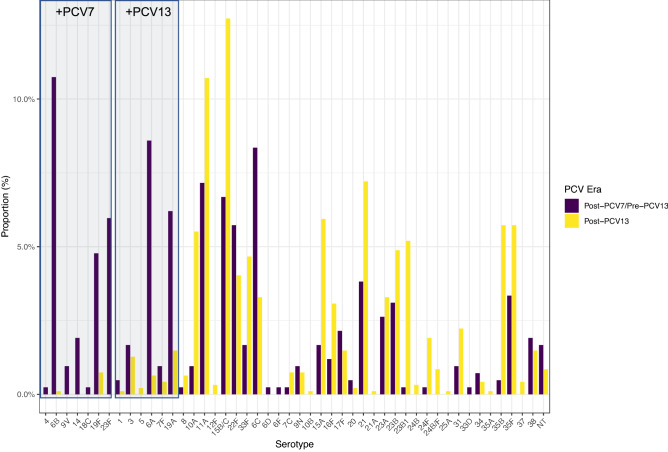
Differences in serotype proportion between the post-PCV7/pre-PCV13 era (purple) and post-PCV13 era (yellow). All PCV serotypes show a marked decrease due to PCV implementation, aside from serotype 3 which has also decreased but to a lesser extent. Notable exceptions to the increase in NVTs are 22F and 6C which have also decreased in the post-PCV13 era, although this followed earlier expansions.

**Figure 7:**
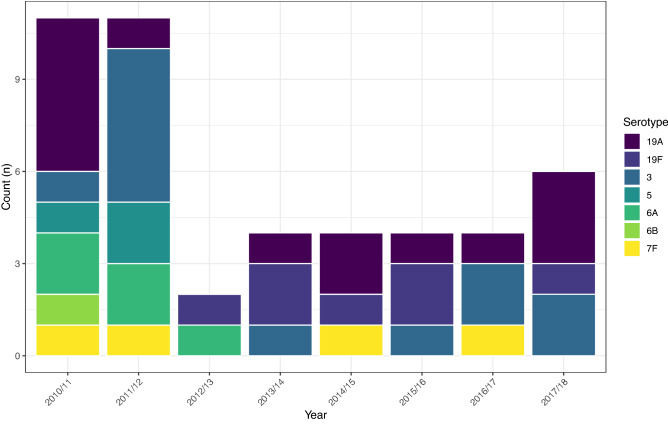
Isolation of PCV-type serotypes following PCV13 introduction in 2010. Serotypes 5, 6B, 6A have not been isolated since 2012/13. In recent years, 19A and 3 have been the most frequently isolated PCV serotypes (n = 7 and n = 5 respectively).

This change was apparent when examining the post-PCV7/pre-PCV13 and post-PCV13 proportion of each serotype individually as shown in Fig. 6. Carriage of all PCV serotypes decreased substantially following PCV7 and/or PCV13 introduction. The exception was serotype 3 where the relative frequency was similar between eras (pre-PCV13 n = 7, 1.67%; post-PCV13 n = 13, 1.27%). NVTs showed generally the reverse with increases in frequency post-PCV introduction. Notable exceptions were 22F and 6C which decreased in the post-PCV period in more recent years. Since 2010/11 n = 47 isolates of a PCV7 or PCV13 serotype have been isolated (Fig. 7) and have included serotypes 6B and 19F from PCV7 and 3, 5, 6A, 7F and 19A from PCV13. No carriage of 5, 6B or 6A has been seen since 2012/13. Single occurrences of 7F carriage occurred in 2014/15 and 2016/17, however 19A and 3 were isolated in most years (n = 7 and n = 5 respectively since 2014/15 inclusive).

**Figure 8:**
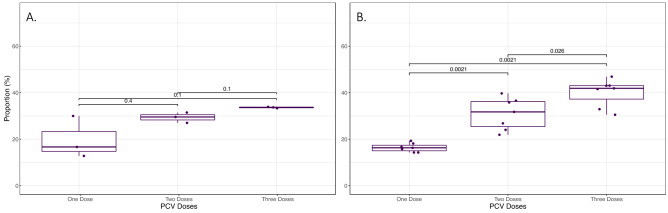
Impact of the estimated number of PCV doses on pneumococcal carriage for PCV7 (**A**.) and PCV13 (**B**.). Carriage as a proportion of number of children within each dose category was significantly higher as dose number increased for PCV13 but not PCV7. Error bars represent 95% CI. Significance values shown are from Kruskal–Wallis comparison of means.

The impact of PCV dose was examined in relation to carriage (Fig. 8). Again, by examining the post-PCV7/pre-PCV13 (Fig. 8A) and post-PCV13 (Fig. 8B) eras separately, a statistically significant increase of carriage in children expected to have received multiple doses was observed: one vs. three doses *p* = 0.0021, one vs. two doses *p* = 0.0021, and two vs. three doses *p* = 0.026. Although a similar trend for PCV7 was observed, smaller counts meant this was not significant.

**Figure 9:**
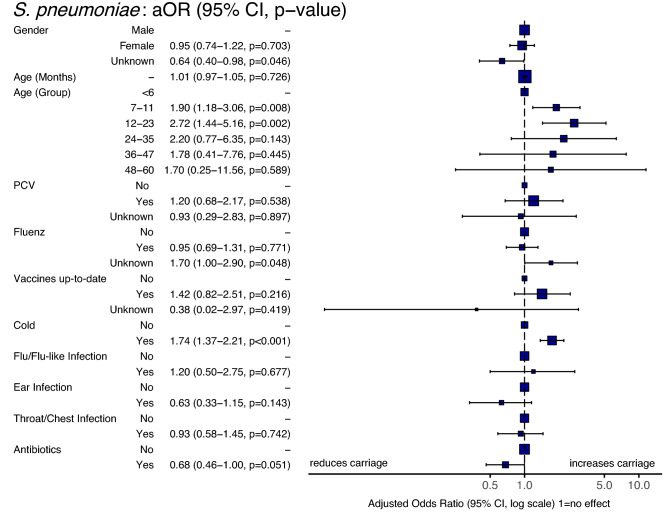
Forest plot showing variables associated with pneumococcal carriage. Odds were calculated using a multivariable logistic regression model. Participants reporting a cold in the previous 30 days were significantly more likely to carry *S. pneumoniae* (OR 1.73:1.36–2.21, *p* < 0.001). Age groups 7–11 and 12–23 months were also more likely to be pneumococcal carriers relative to those < 6 months of age.

The odds of *S. pneumoniae* carriage was examined in relation to age, gender, vaccinations, illness in the 30-days prior to swabbing and antibiotic use in the same period (Fig. 9). The impact of increasing age was seen with both those aged 7–11 and 12–23 months having increased odds for carriage (1.90, 95%CI 1.18–3.06, *p* = 0.008 & 2.72, 95%CI 1.44–5.16, *p* = 0.002 respectively) relative to those < 6 months. Those that reported a cold-like illness in the previous 30 days were significantly more likely to be culture positive for pneumococci (OR 1.74, 95%CI 1.37–2.21, *p* < 0.001). In contrast, those that had received antibiotics were less likely to carry *S. pneumoniae* (0.68, 95%CI 0.46–1.00) although at *p* = 0.051 this did not pass the significance threshold. No other significant relationships were observed.

## Discussion

Monitoring the epidemiology of *S. pneumoniae* is a continuing requirement to safeguard against shifts that might negate vaccine efficacy, or to highlight expansions of disease associated NVTs. These important data may then be used to inform strategies for the design and implementation of increased valency vaccines. Here we present twelve years of data from the unique Southampton pneumococcal carriage study; the only cross-sectional, annual paediatric surveillance study that has been running since the introduction of PCV7 in the UK. We show how the pneumococcal serotype epidemiology of asymptomatic carriage in children < 5 years has altered in this defined geographic region.

Although VT serotypes have significantly decreased, our study shows they have not been eliminated from paediatric carriage completely. Analysis of VT carriage shows that PCV7 serotype 19F and PCV13 serotypes 3, 7F and 19A have all been observed post-PCV13 introduction. This is in keeping with carriage studies in the UK^17^ as well as in the USA, where 19A accounted for 5% of pneumococci 5 years after the introduction of PCV13^18^. Here as well, the frequency of serotype 3, although low, remained unchanged^18^. Further, persistence of 19A in carriage has been noted in the Gambia^19^, 19A and 3 in South African mother–child pairs^20^, and 19A, in particular, in Sweden^21^. In the UK serotype 3 still causes 9.4% of all age IPD, with 19A causing 5.6% and 7F causing 1.6%. The persistence of serotype 3 as a major cause of disease has been noted in many other countries as well^22, 23^. Previous work has determined that both limited direct (immunological) protection from childhood immunisations with knock-on effects for herd immunity are the main causes^24, 25^. However, there is a bias towards carriage studies in young children and consequently much less is known about serotype distributions in older children and healthy adults. For example, recently Adler et al. (2019) found serotype 3 to be the most common serotype found in healthy adults during pre-screening for experimental human pneumococcal colonisation (EHPC)^26^. Serotype 3 was also shown to be carried by older children in England^27^ and both serotype 3 and 19A carriage has been seen in older adults^28, 29^. Clearly this may have important implications for our understanding of invasive potential. Nevertheless, our finding that PCV13 has had little impact on paediatric carriage of serotype 3 is an important observation and is in keeping with other data^21, 30^. Given the recent examination of a new clade of serotype 3, one that is more antibiotic resistant^31^, this warrants further investigation to confirm the phylogenomics of these isolates^32^.

The issues of serotype replacement, plagued by unpredictability when comparing national trends^10^, supports the necessity for continued surveillance. Recent data on the increase in IPD caused by NVTs in adults in England and Wales attributed this burden to serotypes 8, 12F and 9N^6^. Elsewhere, serotype 24F has also been highlighted^30^. Whilst all four of these were identified in carriage, the prevalence was low with 24F (n = 19, 1.4%) being the most common followed by 9N (n = 11, 0.8%), 8 (n = 7, 0.5%) and 12F (n = 3, 0.2%). These ranked 18th, 20th, 23rd and 27th respectively in terms of the frequency of NVTs. That these rarely carried serotypes accounted for > 40% of IPD in England and Wales in 2016/17 suggests high invasive potential and the need for continued surveillance. In contrast, serotypes 15A and 33F, which ranked 7th and 8th in terms of IPD, were the 6th and 11th most isolated serotypes and show a marked increased between post-PCV7/pre-PCV13 and post-PCV13 eras and suggests burden here is due to increased prevalence as opposed to invasive potential.

Our findings that recent respiratory tract infection was associated with increased risk of pneumococcal carriage has been highlighted previously^33^. Whilst not significant there was an indication that recent antibiotic use was associated with lower odds of carriage, again supported by previous studies^34^. Caution should be exercised when interpreting the findings of vaccination and number of PCV doses with increased carriage as it is difficult to disentangle the increases in carriage associated with age in the first three years of life^35^. The analysis presented here is clouded further by having to infer dose number from age as opposed to having immunisation dates for each participant.

The strength of this work is the considerable time over which pneumococcal carriage has been monitored and the annual nature of this surveillance. Consequently, we have been able to examine the impacts of the introduction of both PCV7 and PCV13, making important observations on, for example, the expansion of pneumococcal clones of serotype 6C and 22F^36, 37^. It is also prudent to consider that these data provide important baselines against which to assess changes in vaccine schedule and new formulations. Nevertheless, there are several important limitations to this study. Firstly, any inference to national carriage epidemiology is just that, an inference. Community-level serotype prevalence from a proxy-population will naturally be a poorer substitute for national surveillance and will be hampered by potential geographic and demographic biases. In addition, the analysis of only one isolate from each culture positive individual ignores the impact of colonisation density and multi-serotype carriage as shown elsewhere^38, 39^. Clearly, there are further data such as immunisation dates, co-carriage of other pathobionts and/or viruses that could be collected. Indeed some has been (co-carriage for example), however, the data presented here are the most complete for this twelve-year period i.e., since the initial study design in 2006/07. Finally, the cross-sectional nature means that changes in an individual’s carriage of serotypes over time are missed.

In conclusion we have shown that there is a continued circulation of serotypes 19A and 3 eight years after the introduction of PCV13. Moreover, we have demonstrated that carriage of NVT serotypes, which are now causing significant concern in IPD, are infrequently carried in our paediatric population.

## Supplementary Information

Supplementary Information 1.

Supplementary Information 2.

## Data Availability

All sequencing data (fastqs) has been deposited in the European Nucleotide Archive under study accession PRJEB2417 (Whole genome sequencing of carried *Streptococcus pneumoniae* during the implementation of pneumococcal conjugate vaccines in the UK).
