# Supplementary material for: Changes in serotype prevalence of Streptococcus pneumoniae in Southampton, UK between 2006 and 2018
Source: Sci Rep. 2022 Aug 3;12:13332. doi: 10.1038/s41598-022-17600-6 (PMC9349173; doi:10.1038/s41598-022-17600-6)
Supplement: Supplementary file 1 — Supplementary Information 1. [file 41598_2022_17600_MOESM1_ESM.docx]

Supplementary Table 1: Per year recruitment number with mean age and gender demographics with S. pneumoniae culture positivity.

|  | **1**  **(n = 324)** | **2**  **(n = 373)** | **3**  **(n = 327)** | **4**  **(n = 397)** | **5**  **(n = 288)** | **6**  **(n = 331)** | **7**  **(n = 223)** | **8**  **(n = 315)** | **9**  **(n = 351)** | **10**  **(n = 524)** | **11**  **(n = 441)** | **12**  **(n = 499)** |
| --- | --- | --- | --- | --- | --- | --- | --- | --- | --- | --- | --- | --- |
| **Age (months)** |  |  |  |  |  |  |  |  |  |  |  |  |
| min | 0.5 | 1 | 1.25 | 0.25 | 0.75 | 0.5 | 0 | 0 | 1 | 0 | 0 | 0.5 |
| max | 60 | 59 | 59 | 56 | 60 | 59 | 55.25 | 58 | 59.75 | 59 | 59.75 | 59 |
| mean (sd) | 20.67 ± 14.59 | 25.24 ± 15.15 | 25.10 ± 15.09 | 14.98 ± 12.35 | 20.99 ± 16.43 | 21.05 ± 15.85 | 17.41 ± 11.53 | 21.42 ± 15.27 | 20.47 ± 16.08 | 19.54 ± 15.50 | 20.90 ± 16.59 | 18.41 ± 14.49 |
| **Gender** |  |  |  |  |  |  |  |  |  |  |  |  |
| Male (%) | 179 (55) | 205 (55) | 150 (46) | 191 (48) | 161 (56) | 197 (60) | 124 (56) | 168 (53) | 188 (54) | 255 (49) | 221 (50) | 274 (55) |
| Female (%) | 124 (38) | 148 (40) | 145 (44) | 176 (44) | 109 (38) | 117 (35) | 80 (36) | 118 (37) | 142 (40) | 214 (41) | 190 (43) | 178 (36) |
| Unknown (%) | 21 (6) | 20 (5) | 32 (10) | 30 (8) | 18 (6) | 17 (5) | 19 (9) | 29 (9) | 21 (6) | 55 (10) | 30 (7) | 47 (9) |
| ***S. pneumoniae*** |  |  |  |  |  |  |  |  |  |  |  |  |
| Positive (%) | 104 (32) | 104 (28) | 102 (31) | 111 (28) | 100 (35) | 104 (31) | 78 (35) | 101 (32) | 133 (38) | 190 (36) | 144 (33) | 158 (32) |
| Negative (%) | 220 (68) | 269 (72) | 225 (69) | 286 (72) | 188 (65) | 227 (69) | 145 (65) | 214 (68) | 218 (62) | 334 (64) | 297 (67) | 341 (68) |
