# Supplementary material for: Changes in serotype prevalence of Streptococcus pneumoniae in Southampton, UK between 2006 and 2018
Source: Sci Rep. 2022 Aug 3;12:13332. doi: 10.1038/s41598-022-17600-6 (PMC9349173; doi:10.1038/s41598-022-17600-6)
Supplement: Supplementary file 2 — Supplementary Information 2. [file 41598_2022_17600_MOESM2_ESM.pdf]

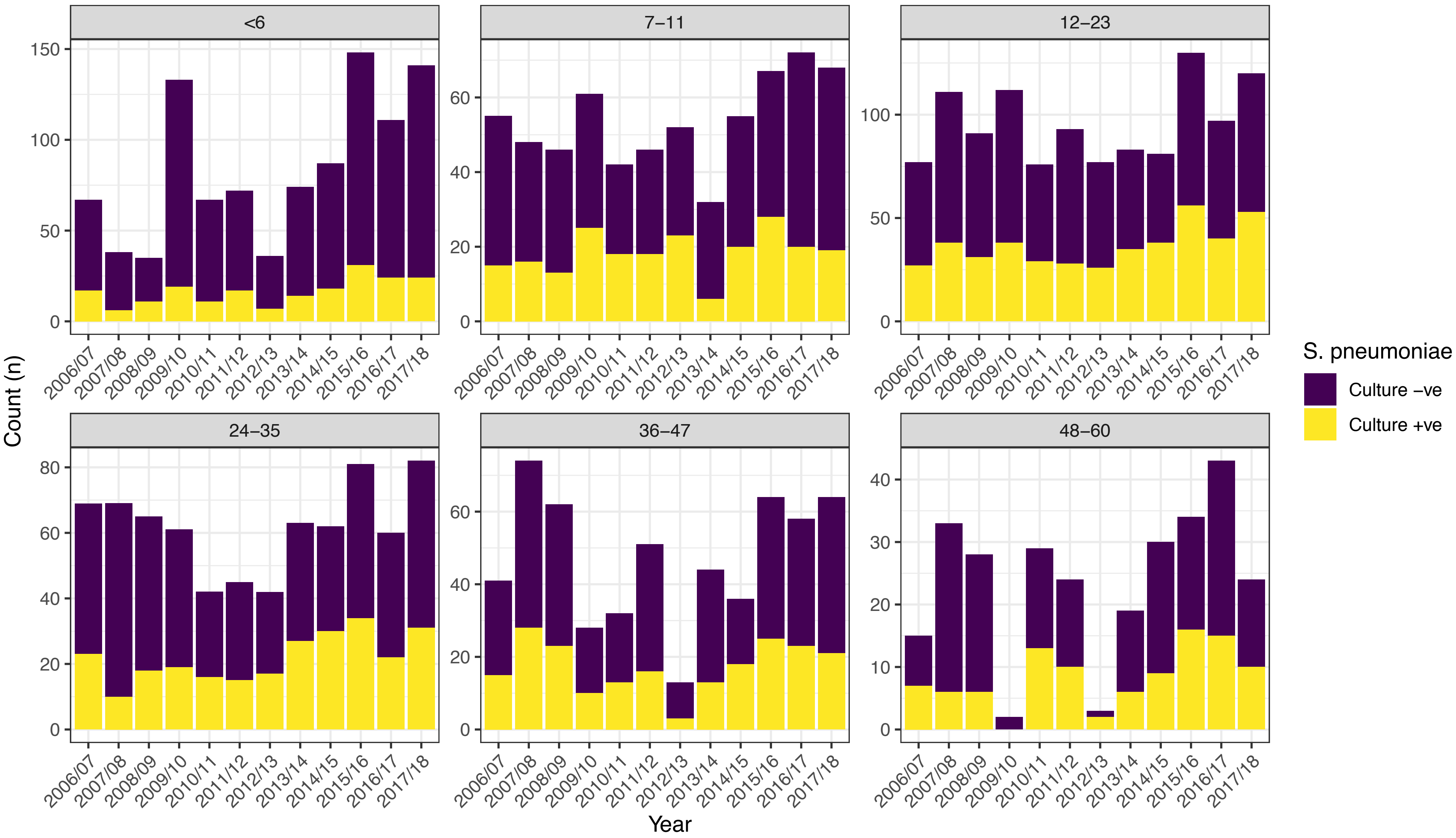

Supplementary Figure 1: Carriage by age and year of study. The number of children who carried pneumococci are shown in yellow and those that did not in purple.
